# A novel approach to measure the contribution of matrix metalloproteinase in the overall net proteolytic activity present in synovial fluids of patients with arthritis

**DOI:** 10.1186/ar2014

**Published:** 2006-07-19

**Authors:** Nathalie Simard, Gilles Boire, Artur J de Brum-Fernandes, Yves St-Pierre

**Affiliations:** 1 INRS-Institut Armand-Frappier, University of Québec, Laval, Québec, Canada; 2 Division of Rheumatology, Department of Medicine, Centre hospitalier universitaire de Sherbrooke, Université de Sherbrooke, Québec, Canada

## Abstract

Despite decades of research, only a very limited number of matrix metalloproteinase (MMP) inhibitors have been successful in clinical trials of arthritis. One of the central problems associated with this failure may be our inability to monitor the local activity of proteases in the joints since the integrity of the extracellular matrix results from an equilibrium between noncovalent, 1:1 stoichiometric binding of protease inhibitors to the catalytic site of the activated forms of the enzymes. In the present work, we have measured by flow cytometry the net proteolytic activity in synovial fluids (SF) collected from 95 patients with osteoarthritis and various forms of inflammatory arthritis, including rheumatoid arthritis, spondyloarthropathies, and chronic juvenile arthritis. We found that SF of patients with inflammatory arthritis had significantly higher levels of proteolytic activity than those of osteoarthritis patients. Moreover, the overall activity in inflammatory arthritis patients correlated positively with the number of infiltrated leukocytes and the serum level of C-reactive protein. No such correlations were found in osteoarthritis patients. Members of the MMP family contributed significantly to the proteolytic activity found in SF. Small-molecular-weight MMP inhibitors were indeed effective for inhibiting proteolytic activity in SF, but their effectiveness varied greatly among patients. Interestingly, the contribution of MMPs decreased in patients with very high proteolytic activity, and this was due both to a molar excess of tissue inhibitor of MMP-1 and to an increased contribution of other proteolytic enzymes. These results emphasize the diversity of the MMPs involved in arthritis and, from a clinical perspective, suggest an interesting alternative for testing the potential of new protease inhibitors for the treatment of arthritis.

## Introduction

Degradation of various macromolecules composing the extracellular matrix is a hallmark of most forms of arthritis. These changes are mediated by an excess of activity resulting from an increased expression of the active form of the proteases, and/or from an altered equilibrium between the proteases and their inhibitors in inflamed synovial membrane and synovial fluids (SF) [1-4]. This provided a rationale for the development of broad-spectrum matrix metalloproteinase (MMP) inhibitors as a new class of drugs [5,6].

The failure of these MMP inhibitors in clinical trials may at least in part be explained by the fact that the magnitude and specificity of protease activity changes were not directly measured. Indeed, conventional assays used to monitor the presence of MMPs in SF, such as ELISA and zymography, do not provide a direct measurement of their net proteolytic activity (NPA). The NPA depends on the activation status of the enzyme and on the balance between active proteases and endogenous protease inhibitors, such as tissue inhibitors of MMPs (TIMPs) [7,8]. Hence, it is the equilibrium between active proteases and inhibitors that determines the level of contribution of a specific protease to cartilage degradation, and not simply its expression level. This may explain why, while MMP-3 levels in SF of rheumatoid arthritis (RA) patients are extremely high [3,9], depletion of MMP-3 in animal models does not prevent cleavage of aggrecan, nor does it prevent or reduce cartilage destruction observed in specific forms of arthritis [10-12].

This lack of causal relationship between the expression levels of specific MMPs and cartilage destruction may explain the limited success of MMP inhibitors in clinical trials, and emphasizes the importance of measuring the NPA of proteases [13]. In the present work, using a flow-cytometric-based assay that directly measures the NPA of MMPs in SF, we provide new insights into the overall contribution of these enzymes to the proteolytic activity in arthritic joints.

## Materials and methods

### Reagents

Gelatin and fluorescein isothiocyanate (FITC) were obtained from Sigma (St Louis, MO, USA). Polystyrene microspheres were purchased from Polysciences (Warrington, PA, USA). The blocking antibody specific for human MMP-9 was obtained from Santa Cruz (Santa Cruz, CA, USA), and the recombinant MMPs and their inhibitors were from Calbiochem (San Diego, CA, USA). The human TIMP-1 ELISA kit was purchased from R&D Systems (Minneapolis, MN, USA).

### Sampling of synovial fluids and sera

Patients evaluated by rheumatologists from the Rheumatology Division of the Centre Hospitalier Universitaire de Sherbrooke were asked to participate in this study. Criteria for admission to the study were the clinical indication for a therapeutic and/or diagnostic arthrocentesis of one or several articulations and a willingness to participate in the study. No exclusions were made on any basis other than an inability or unwillingness to give informed consent or the contamination of the SF by blood during arthrocentesis.

A total of 95 samples of SF were used: 15 samples from patients with established RA defined according to the 1987 American College of Rheumatology criteria [14], 14 samples from patients with undifferentiated or recent-onset polyarthritis, 26 samples from osteoarthritis (OA) patients, 13 samples from patients with crystal-induced arthritis, and 27 samples from patients with other noninfectious inflammatory arthritis (IA), such as ankylosing spondylitis and reactive arthritis. A patient with features of more than one rheumatic disease (e.g. OA and crystal-induced arthritis) was classified as having the disease that clinically predominated at the time of arthrocentrisis.

After informed consent, venous blood (5 ml) and 2 ml SF collected within 1 hour were sent to the appropriate clinical laboratories. A differential blood cell count was performed on the blood samples, and appropriate microbiologic studies to exclude infectious arthritis, a differential nucleated cell count, and crystal identification were performed on the SF. Venous blood (10 ml) and SF in excess of the 2 ml needed for the clinical purposes described were also collected. Sera were stored at -80°C until used. SF were clarified by centrifugation at 1500 × *g *for 15 min and were stored in aliquots at -80°C for subsequent analysis.

The study included SF from patients with and without medication. Patients receiving medication were given nonsteroidal anti-inflammatory drugs, either alone or in combination with glucocorticosteroids (prednisone), or were given disease-modifying antirheumatic drugs, such as methotrexate.

This study was conducted with the approval of the ethics review board of the Centre Hospitalier Universitaire de Sherbrooke.

### Detection of matrix metalloproteinases by zymography

The gelatinase activity of SF was determined as previously described [15]. The gelatinolytic activity was measured in arbitrary units by quantitative analysis of a negatively stained band using computerized image analysis (Image Quant 5.0; Molecular Dynamics, Sunnyvale, CA).

### Measure of the net proteolytic activity in synovial fluids from arthritic patients

The NPA was measured by fluorescent-activated substrate conversion (FASC) [16] with the following modifications.

#### Fluorescent labelling of substrates

Denatured collagen was dissolved at a final concentration of 2 mg/ml in carbonate buffer (pH 9.2). FITC (dissolved in dimethylsulfoxide at 5 mg/ml) was added to the substrate (20 μl/mg). Labelling was carried out for 2 hours at room temperature. Free FITC molecules were removed by chromatography on PD-10 columns (Pharmacia, Uppsala, Sweden) using PBS (pH 7.4) as the eluent buffer. FITC is a small molecule, thereby minimizing the steric hindrance around putative cleavage sites. It is also easily conjugated and compatible with most commercial flow cytometers.

#### Microsphere coating

Polystyrene microspheres, 4.5 μm in diameter, were incubated overnight at room temperature with the FITC-conjugated substrate (15 μg/ml in PBS, pH 7.4) to allow noncovalent adsorption. The microspheres were then washed three times in PBS (pH 7.4) containing 0.05% sodium azide. Microspheres were kept at 4°C in PBS in the dark and were resuspended by gentle vortexing before use.

#### FASC assay and flow cytometric analysis

While the pH of normal synovial fluid is approximately 7.4, previous reports have shown that its value varies slightly in various inflammatory conditions [17] – with a tendency to decrease with conditions associated with an increasing leukocyte count [18]. Since MMPs are neutral endopeptidases and are highly active over a wide range of pH values, including lower acidic pH values, we have chosen to measure the overall activity at pH 7.4.

The enzymatic reactions were thus performed at 37°C for 18 hours in a final volume of 50 μl digestion buffer (20 mM Tris–HCl, 150 mM NaCl, 10 mM Ca^2+^, 0.05% NaN_3_; pH 7.4). The samples contained 10 μl SF at various dilutions and 5 × 10^4 ^(5 μl) FITC-labelled substrate-coated microspheres. The volume was completed to 50 μl with digestion buffer. The reaction was stopped by adding 300 μl reading buffer (pH 9.5). To titrate the proteolytic activity in arthritic SF, dilutions ranging from 1:50 to 1:6250 (v/v) were prepared. Trypsin was used as a positive control for enzymatic activity.

Samples were analysed on a Coulter XL-MCL flow cytometer (Hialeah, FL, USA) using standard optics for detection of FITC fluorescence. For each sample, 2000 positive events were analysed.

### Statistical analyses

Data represent the mean ± standard error of the mean. The statistical significance of differences between means was determined by either Student's *t *test for comparisons between two groups or Spearman rank order correlations. *P *< 0.05 was accepted as statistically significant.

## Results

### Measure of net proteolytic activity in synovial fluids

To quantitatively assess the actual level of proteolytic activity in fluids, we used the FASC assay [16]. Fluorescent-labelled large polypeptides derived from denatured collagen are immobilized on polystyrene microspheres. These microspheres are then incubated at 37°C for 18 hours with serial dilutions of SF, and the proteolytic activity is monitored by the decrease of the fluorescence emitted by the microspheres via flow cytometry.

**Figure 1:**
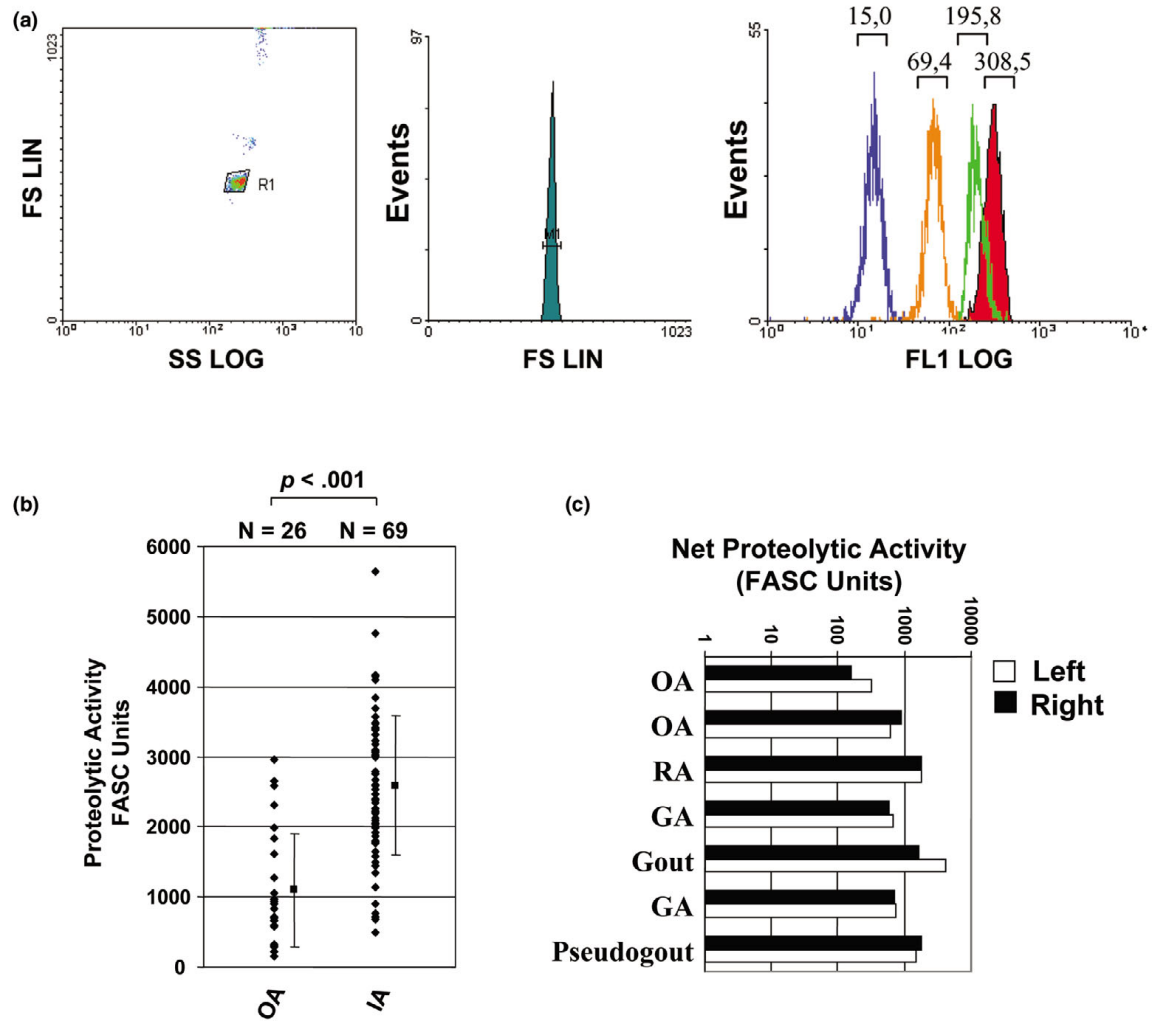
Proteolytic activity in synovial fluid of patients with osteoarthritis and inflammatory arthropathies. **(a) **Typical two-parameter histogram (left) with forward-angle light scatter (FS) and side scatter (SS) (*y *and *x *axes, respectively), used to position the window on microspheres (one-parameter histogram, middle) and to minimize the interference with debris. To determine the proteolytic activity in synovial fluids (SF), fluorescein isothiocyanate-labelled polypeptides derived from denatured collagen (gelatin) were coated on polystyrene microspheres (shaded) and incubated with serial dilution of synovial fluid. LIN, Linear; LOG, Logarithmic. The mean fluorescent intensity at the top of each peak is then measured on 1,000–5,000 events (right). **(b) **Comparison of the level of proteolytic activity found in SF obtained from 26 osteoarthritis (OA) patients and 69 patients with inflammatory arthritis (IA), including patients with established rheumatoid arthritis (RA) or undifferentiated or recent-onset polyarthritis, patients with crystal-induced arthritis, or patients with other noninfectious IA, such as ankylosing spondylitis and reactive arthritis. FASC, fluorescent-activated substrate conversion. **(c) **Proteolytic activity in SF collected from the left and right knees of arthritic patients on the same day. The results are representative of at least two independent experiments. GA, Gout arthritis.

For the analyses, a two-parameter histogram with the forward-angle light scatter and side scatter presented on the *x *axis and the *y *axis, respectively, is used to position the window on substrate-coated microspheres, thereby minimizing interference with debris (Figure 1a, left panel). The NPA in the clinical sample is then obtained by measuring the mean fluorescence intensity resulting from the loss of fluorescent substrate following incubation of the microspheres with serial dilutions of fluids at 37°C for 18 hours (Figure 1a, right panel).

The microspheres are analysed in a very small volume as they pass through the flow cytometers' laser beam, and therefore free fluorescent molecules (cleaved substrates) do not interfere with the measure of fluorescence, obviating the requirement for washing the microspheres after incubation with the samples prior to the flow cytometric data acquisition. This approach therefore allows one to rapidly and accurately quantify the presence of active extracellular proteases in human body fluids upon cleavage of the peptide bond. Previous studies have shown that large polypeptides derived from denatured collagen are a convenient source of substrates to measure the contribution of each of the four classes of proteases, including serine, cysteine, and aspartic peptidases [16,19] (data not shown).

In the first series of experiments, we quantitated the levels of activity in SF from 95 patients with various forms of IA. To accurately compare the net proteolytic activity between samples, the activity was determined by testing serial dilutions (1:50–1:6,250); the activity is expressed as FASC units, 1 FASC unit being defined as the reciprocal dilution that resulted in 40% cleavage of the fluorescent substrate. We found that proteolytic activity was present at high levels in all synovial samples, with IA patients exhibiting significantly higher levels of NPA than OA patients (mean ± SEM, 2,589 ± 994 units/ml for IA versus 1,097 ± 810 units/ml for OA; *P *< 0.001) (Figure 1b). Moreover, we also found that the titre of activity varied greatly among the different samples, ranging from approximately 300–500 units in most OA cases to more than 3,500–5,000 units in SF obtained from the knee of patients with spondyloarthropathies.

Interestingly, we also found that the NPA was similar in the fluids collected from two different joints from the same patient on the same day (Figure 1c), independently of the type of arthritis and the level of proteolytic activity. This suggests that, in the case of arthritis with involvement of several joints, systemic factors play a dominant role in controlling the local proteolytic activity. Finally, we did not detect any significant activity in normal SF obtained from healthy controls (<10 units/ml) (data not shown). No enzymatic activity was similarly found in the serum obtained from healthy subjects, or in serum of patients with any form of arthropathies, consistent with previous reports showing that human serum contains high concentrations of endogenous protease inhibitors [20].

These results demonstrate that this approach can be used to quantify the NPA resulting from the overall repertoire of proteases present in biological fluids collected from human patients.

### Proteolytic activity in synovial fluids of inflammatory arthritis patients correlates with the inflammatory state of the disease

**Figure 2:**
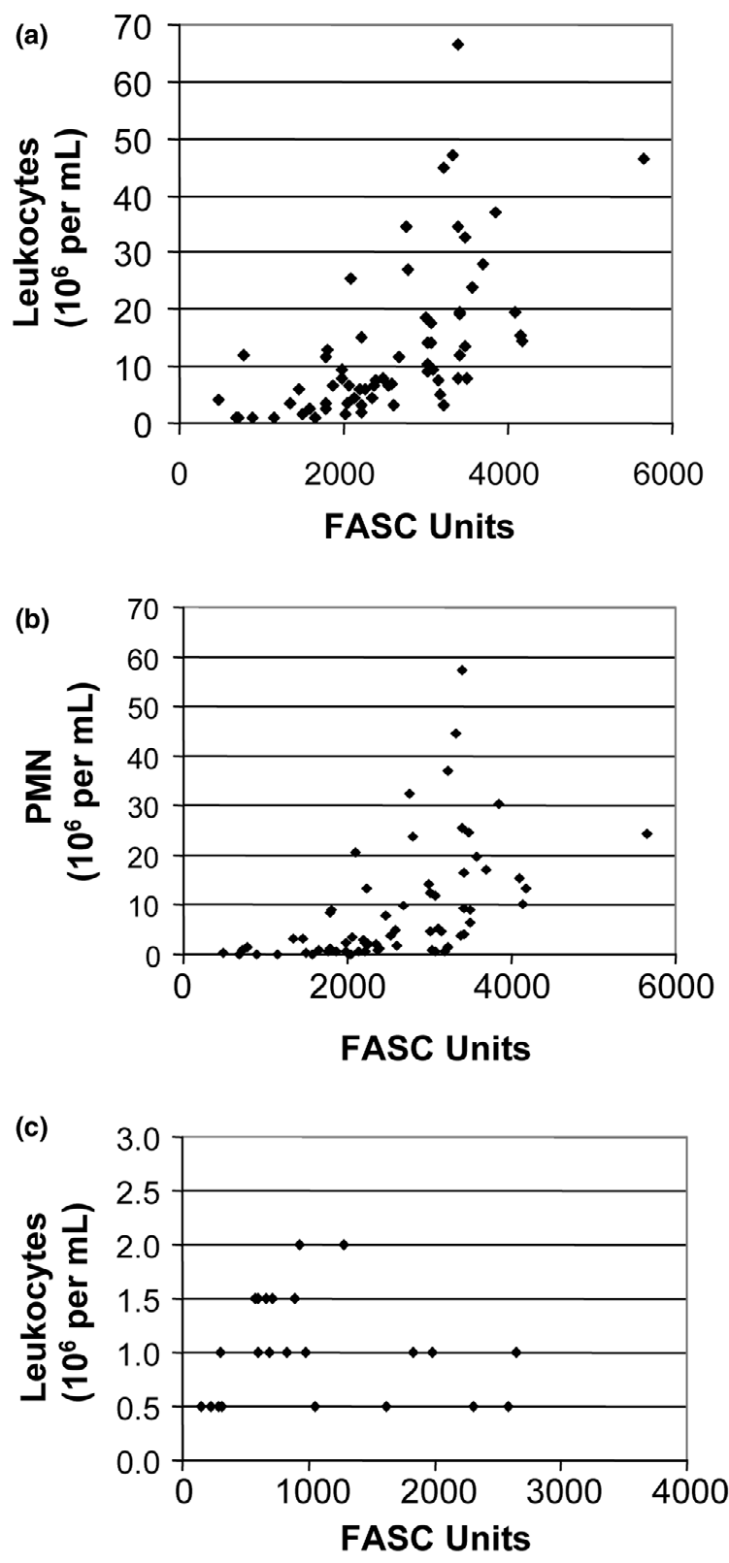
Correlation between the level of proteolytic activity found and the number of infiltrating leukocytes. Positive correlation between the net proteolytic activity (NPA) and the number of **(a) **infiltrating leukocytes (*r*_s _= 0.710, *P *< 0.001) or **(b) **polymorphonuclear cells (PMN) (*r*_s _= 0.696, *P *< 0.001) in synovial fluids of patients with inflammatory arthritis. **(c) **No such correlation between the NPA and the number of infiltrating leukocytes was observed in osteoarthritis (OA) patients. Each point represents one sample. The correlation was calculated using Spearman's rank correlation coefficient for each test. FASC, fluorescent-activated substrate conversion.

Extracellular proteases are secreted in SF by infiltrating inflammatory cells and/or by stromal cells stimulated by inflammatory cytokines [21,22]. To assess whether inflammation does correlate with the proteolytic activity in arthritic joints, we first compared the NPA with the number of infiltrating leukocytes in SF of IA patients. Our results showed that NPA correlated positively (*r*_s _= 0.710, *P *< 0.001) with the number of infiltrated leukocytes (Figure 2a) or with the number of infiltrated polymorphonuclear cells (r_s _= 0.696, *P *< 0.001) (Figure 2b). Weaker but significant correlations were also observed with the number of monocytes (*r*_s _= 0.422, *P *< 0.001) and lymphocytes (*r*_s _= 0.405, *P *= 0.001) (data not shown). No such correlation between the NPA and the number of infiltrating leukocytes was observed in the case of patients with OA (Figure 2c).

To further assess how the proteolytic activity correlates with the inflammation, we examined C-reactive protein, a well-known inflammatory marker [23]. We found that C-reactive protein levels correlated positively (*r*_s _= 0.405, *P *= 0.009) with the NPA in IA patients. The association between C-reactive protein levels and the NPA was more striking (*r*_s _= 0.899, *P *< 0.001) in patients with undifferentiated polyarthritis. In contrast, no such correlation was observed between C-reactive protein levels and the NPA in fluids of OA patients, or between the NPA and sex or age of the patient (data not shown).

### The expression level of MMP-9 is not indicative of the net proteolytic activity

**Figure 3:**
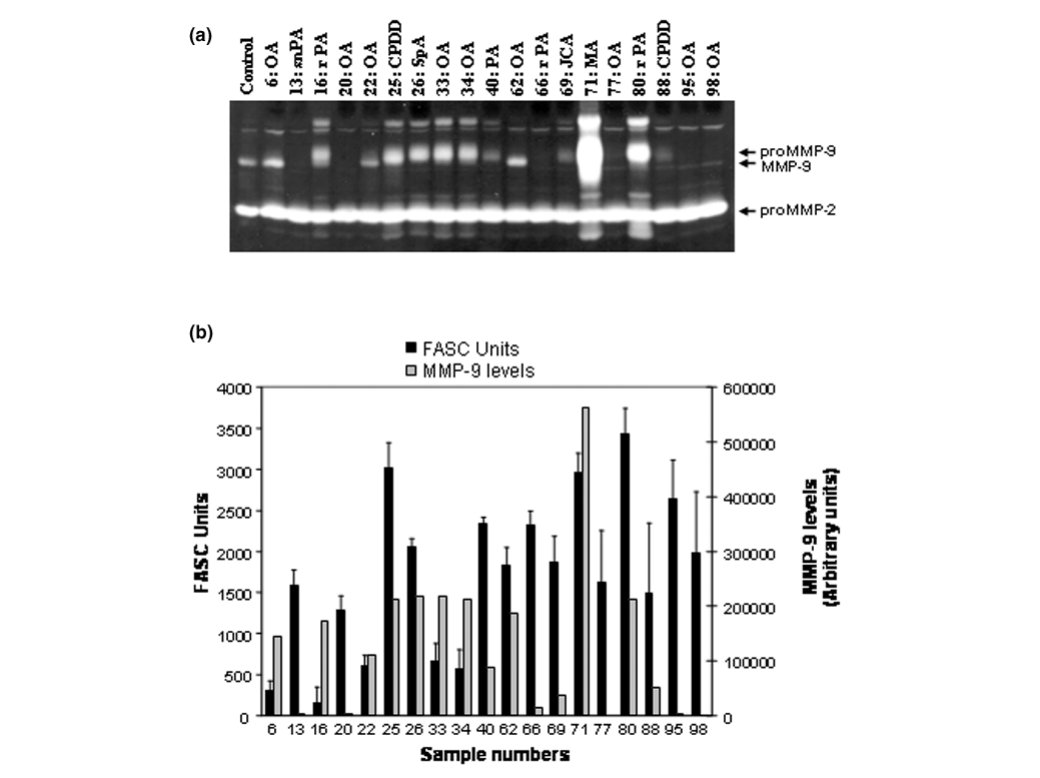
Zymographic analysis of MMP-2 and MMP-9 levels in arthritic synovial fluids. **(a) **Secretion of MMP-2 and MMP-9 was assessed by gelatin zymography using standard procedures. Aliquots of 10 μl synovial fluids (SF) (diluted 1:10) were loaded into each lane. OA, osteoarthritis; snPA, seronegative polyarthritis; rPA, seropositive rheumatoid polyarthritis; CPDD, calcium phosphate deposition disease; SpA, spondyloarthropathy; PA, polyarthritis; JCA, juvenile chronic arthritis; MA, microcrystalline arthritis. **(b) **Semiquantitative measures of the proMMP-9 level were carried out by densitometric analysis (scored as arbitrary units) and compared with the net proteolytic activity (NPA) found in the same SF. No correlations between the NPA and the level of the 225 kDa form of MMP-9 or with that of its active form (92 kDa) were observed (data not shown). FASC, fluorescent-activated substrate conversion. As a positive control, an aliquot of supernatant of HT1080 cell culture containing both MMP2 and MMP-9 gelatinases was used. The results were obtained using duplicates and are representative of two independent experiments. Error bars represent the mean ± standard error of the mean.

Excessive secretion of members of the MMP family is the hallmark of several inflammatory disorders, including arthritis. MMP-9, in particular, has been implicated in the degradation and damage of articular cartilage in RA and OA [2-4]. We thus examined the levels of MMP-9 in SF from 19 patients with different forms of arthritis, including RA, OA, chronic juvenile arthritis, and other forms of arthritis, and compared them with the levels of the NPA. The level of MMP-9 secretion was very high in some fluids, although a significant heterogeneity was observed among the samples (Figure 3a). These results were not surprising since the levels of MMP-9 in SF have been shown to vary according to several factors, such as the disease state, the local concentration of inflammatory cytokines, and the number of infiltrating leukocyte subpopulations [24,25]. Most importantly, we found that the level of MMP-9 did not correlate with the level of the NPA (Figure 3b). This was particularly evident in samples 13, 20, 66, 95, and 98, which had a very high level of NPA but very low levels of MMP-9.

### Implication of matrix metalloproteinases in proteolytic reactivity found in synovial fluids of RA patients

To further assess the contribution of MMP-9 and other members of the MMP family in the NPA of patients with IA, we used two hydroxamic acid-based broad-spectrum MMP inhibitors (inhibitor II and inhibitor III). These inhibitors have been shown to be selective for two relatively distinct repertoires of MMPs – whereas inhibitor II inhibits MMP-1, MMP-3, MMP-7 and MMP-9, inhibitor III blocks MMP-1, MMP-2, MMP-3, MMP-7 and MMP-13 [26].

**Figure 4:**
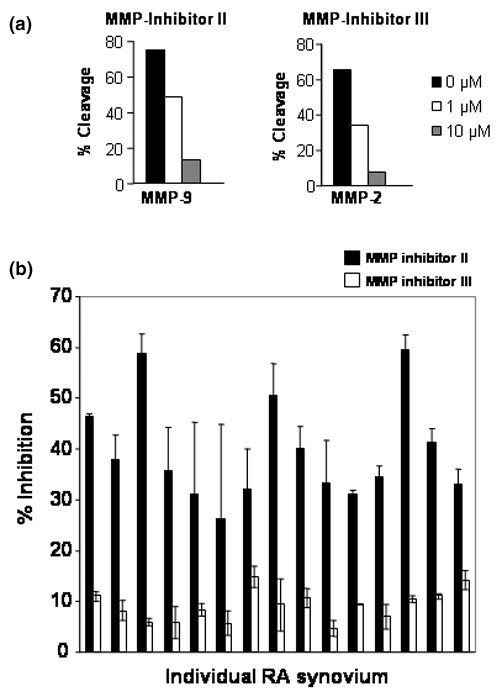
Effect of hydroxamate-based inhibitors on arthritic synovial fluids. **(a) **Detection of the inhibitory effect of the matrix metalloproteinase (MMP) inhibitors (inhibitor II and inhibitor III) at different doses by fluorescent-activated substrate conversion using recombinant human MMP-2 and MMP-9. Briefly, recombinant MMP-2 and MMP-9 were incubated with microspheres coated with fluorescein isothiocyanate-labelled polypeptides (derived from denatured collagen) at 37°C. The percentage of cleavage, measured by the loss of fluorescence, was measured by flow cytometry using standard optics for fluorescein (using a 525 nm bandpass filter). **(b) **Effect of the hydroxamate inhibitors (used at 10 μM) on rheumatoid arthritis (RA) synovial fluids. Each bar represents one sample. The results were obtained using duplicates and are representative of two independent experiments. Error bars represent the mean ± standard error of the mean.

All these MMPs are also elevated in SF during arthritis. For instance, MMP-1, which is produced primarily by synovial cells, and MMP-13, a product of the chondrocytes, have predominant roles because they are rate limiting in the process of collagen degradation [27,28]. The efficiency of these two inhibitors to block the enzymatic activity of MMPs at different doses was first confirmed using recombinant MMPs (Figure 4a). When testing the activity using SF of patients with RA, we found that while the percentage of inhibition of the NPA in the presence of inhibitor III rarely exceeded 10–15%, the level of inhibition obtained using inhibitor II was significantly higher, ranging from 25% to 60% (Figure 4b). These results indicate that MMPs contribute to a large extent to the NPA found in SF, but that the repertoire of MMPs varies considerably among patients.

**Figure 5:**
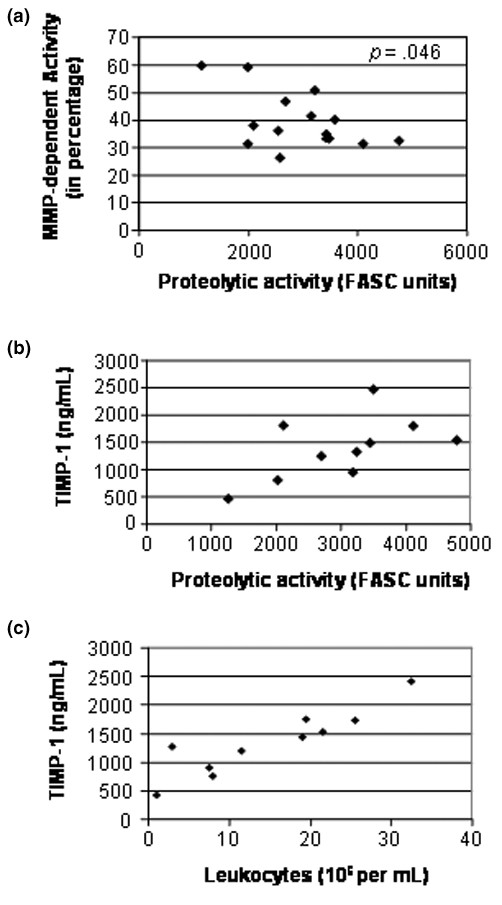
Levels of TIMP-1 in synovial fluid of rheumatoid arthritis patients. **(a) **Inverse correlation between the matrix metalloproteinase (MMP)-dependent proteolytic activity and the level of proteolytic activity in synovial fluids of rheumatoid arthritis (RA) patients (*r *= -0.521, *P *= 0.046). **(b) **Correlation between the level of TIMP-1 and the net proteolytic activity (*r *= 0.592, *P *= 0.07). **(c) **Positive correlation between the level of TIMP-1 and the number of infiltrated leukocytes (*r *= 0.910, *P *< 0.001). The levels of TIMP-1 in synovial fluids were measured by commercial ELISA. The contribution of MMPs to the proteolytic activity was carried out using the hydroxamate inhibitor II, as described in Figure 4. Each point represents one sample. The correlation was calculated using Spearman's rank correlation coefficient for each test. FASC, fluorescent-activated substrate conversion.

Interestingly, however, we found that the effect of the MMP inhibitor II dropped significantly (*P *= 0.046) as the NPA increased (Figure 5a). We then examined whether the decreased contribution of MMPs in those fluids with a high proteolytic activity correlated with an increased TIMP-1 secretion. Our results showed that these fluids had a very high level of TIMP-1 (Figure 5b) that correlated positively with the number of leukocytes (*r *= 0.910, *P *< 0.001) (Figure 5c), suggesting that leukocytes may contribute positively to the formation of TIMP:MMP complexes. No such correlation between TIMP-1 and the proteolytic activity was found in OA patients (*r *= 0.880, *P *= 0.55).

## Discussion

To the best of our knowledge, this is the first study assessing the NPA in SF from patients with different forms of arthritis. Levels of active MMPs in the joint are frequently quantified using immunological methods that do not discriminate between MMPs that are active, that are in their latent form, or that are bound to endogenous protease inhibitors. Our approach clearly showed several results. First, the NPA in patients with IA correlated positively with the number of infiltrated leukocytes. Also, although the expression level of MMP-9 is not indicative of the NPA found in SF, MMPs contribute to a large extent to the proteolytic activity. Third, small molecular weight inhibitors were very effective in inhibiting MMP activity in RA SF, but their effectiveness varied significantly among patients. Finally, and most interestingly, increased TIMP-1 secretion by leukocytes in patients with IA shifted the balance toward an MMP-independent proteolytic activity in those SF with high NPA. Overall, these results stress the importance of monitoring the repertoire of active proteases, not simply the presence of proteases, in patients during the course of their disease and under the influence of treatment. This may represent a *sine qua non *to achieve clinical success in the development of new generations of protease inhibitors with superior clinical efficacy.

Our findings that the NPA is higher in RA than in OA are consistent with previous reports that levels of MMPs and other proteases are higher in RA [2,29,30]. This observation thus supports the idea that inflammation plays an important role in controlling the protease/inhibitor ratio in SF [31], and is consistent with the recent observation of a direct correlation between tumour necrosis factor alpha/MMP production and collagen degradation [32]. Indeed, we found that the NPA in SF correlates positively with the number of infiltrating leukocytes and with blood C-reactive protein levels. This finding suggests that leukocytes control, either directly or indirectly, the overall balance that results in the NPA found in SF – most probably by secreting proteases, or by inducing the secretion of proteases via the secretion of cytokines [33].

Our results showing elevated levels of TIMP-1 in SF with the highest levels of NPA, both of which correlated with the levels of leukocytes, further emphasize the importance of infiltrating leukocytes in controlling the NPA of SF. Our results thus illustrate the need to measure the NPA resulting from the (dis)equilibrium between active proteases and their inhibitors. Other workers have also reported a molar excess of TIMP-1 in SF in animal models of OA, suggesting that matrix degeneration in osteoarthritic joints could be promoted by other proteases such as aggrecanase-1 and aggrecanase-2 [34]. The use of other protease inhibitors specific for cathepsins, elastase, and other types of proteases previously found in SF of RA patients [29,35] will be necessary to determine the contribution of these proteases to the NPA in various diseases.

We used the ability of the flow cytometer to accurately detect different classes of particles, such as microspheres, based upon a physical characteristic such as size and scattering, to develop a simple and reliable method that allows qualitative and quantitative measurements of specific enzymatic reaction using fluorochrome-labelled substrate coated onto polystyrene microspheres [8]. The advantage of our approach is that the fluorochrome-labelled substrates are used in the context of laser flow cytometry, which increases the sensitivity of the assay. Using spheres of multiple sizes, one could also use this assay to perform real-time multiple determinations to determine the repertoire of different active proteases using selective substrates.

Flow cytometers hydrodynamically focus a fluid suspension of particles into a thin stream so that the microspheres flow down the stream in single file and pass through an examination zone. A focused laser light beam illuminates the spheres as they flow through the examination zone, allowing optical detectors within the flow cytometers to measure the fluorescence bound to the microspheres. Because the beads are analysed in a very small volume (about 6 pl) as they pass through the flow cytometers' laser beam, interference from free fluorescent molecules (cleaved substrate) does not interfere with the assay. This design is thus compatible with the use of highly specific and high-affinity inhibitors, such as MMP-specific monoclonal antibodies with neutralizing activity.

Such a measure of net enzymatic activity allows, in the case of proteases, one to specifically account for the presence of enzymatic inhibitors in SF. This is a key issue as the degradation of the tissue architecture and disease state depend on the biological activity of the enzymes; that is, on the ratio of free active enzymes to inactive (inhibitor-bound) enzymes.

In the present article we have provided evidence that this method constitutes a powerful tool to assess the performance of enzyme inhibitors for therapeutic applications. For example, our results with inhibitor II and inhibitor III suggest that MMP-2 and MMP-13 play a dominant role in the proteolytic activity found in the SF of RA patients. One could thus be able to determine which among the available protease inhibitors is best suited to inhibit the proteolytic activity in SF of a given patient. Since we found that MMP-2 is mostly found in its proform in SF, our results favour the implication of MMP-13 Interestingly, MMP-13, which is responsible for cleavage of type II collagen [36,37], aggrecan [38], and fibrinogen [39], has been shown to be increased in RA SF [3] and to be linked to synovial inflammation and bone destruction [40]. Of course, one has to be careful with such conclusion, as it is probable that the repertoire of active proteases will vary with specific subgroups of inflammatory arthritis. For instance, Peake and colleagues recently showed that MMP-13 was not detected in SF or serum of patients with juvenile idiopathic arthritis [41].

Future work is clearly required to establish with more precision the repertoire of active proteases in arthritic SF. The monitoring of activity on a patient may allow adjusting the dosage of a specific inhibitor or, alternatively, exploring whether additional combinatorial treatments are required. From a clinical point of view, therefore, this assay represents an ideal approach for testing the potential of new protease inhibitors aimed at inhibiting disease progression. Failure to do so may explain at least in part the current observation that MMP inhibitors failed in clinical trials. It is probable, however, that other factors may explain the failure of MMP inhibitors in clinical trials. For instance, some members of the MMP family were recently shown to exert an anti-inflammatory activity in some physiological processes or diseases. This hypothesis received strong support from data obtained using genetically engineered MMP-deficient mouse models [42]. Moreover, cleavage of chemokines by MMPs has been shown to generate chemokine receptor antagonists that retain cellular binding affinity while inhibiting the biological activity of the receptor [43]. SDF-1α and other cytokines/chemokines can also be degraded by several members of the MMP family that are constitutively expressed at high levels in a particular tissue, impairing their receptor-binding activity and their ability to mediate chemotaxis [44]. In contrast, other chemokines, such as IL-8, can be proteolytically activated by MMP-9 and MT1-MMP [45].

A more complete understanding of the joint destructive process and of the identity of the relevant proteases are keys to the future development of protease inhibitors in rheumatic diseases. Additional studies are needed to investigate the overall correlation between the types and levels of active MMPs that are found in SF and those MMPs that are found in the inflamed synovial tissues.

## Conclusion

We have been able, using a flow-cytometric-based assay that can directly measure the net proteolytic activity resulting from the balance between the active forms of proteases and the naturally occurring protease inhibitors, to measure for the first time the diversity of the repertoire of active MMPs in arthritic SF. Although the identity of the specific MMPs involved in different types of arthritis remains to be determined, from a clinical point of view this approach represents an interesting tool to test the potential of new protease inhibitors for the treatment of arthritis.

## Abbreviations

ELISA = enzyme-linked immunosorbent assay; FASC = fluorescent-activated substrate conversion; FITC = fluorescein isothiocyanate; IA = inflammatory arthritis; IL = interleukin; MMP = matrix metalloproteinase; NPA = net proteolytic activity; OA = osteoarthritis; PBS = phosphate-buffered saline; RA = rheumatoid arthritis; SF = synovial fluids; TIMP = tissue inhibitor of MMP.

## Competing interests

The authors declare that they have no competing interests.

## Authors' contributions

NS carried out the *in vitro *experiments. YS-P and NS designed the study, carried out the experiments, and drafted the manuscript. GB and AJdBF collected the clinical materials and revised the manuscript. All authors read and approved the final manuscript.
